# BglBricks: A flexible standard for biological part assembly

**DOI:** 10.1186/1754-1611-4-1

**Published:** 2010-01-20

**Authors:** J Christopher Anderson, John E Dueber, Mariana Leguia, Gabriel C Wu, Jonathan A Goler, Adam P Arkin, Jay D Keasling

**Affiliations:** 1 Department of Bioengineering, University of California, Berkeley, CA 94720, USA; 2 QB3: California Institute for Quantitative Biological Research, University of California, Berkeley, CA 94720, USA; 3 Physical Biosciences Division, Lawrence Berkeley National Laboratory, Berkeley, CA 94720, USA; 4 Synthetic Biology Engineering Research Center, University of California, Berkeley, CA 94720, USA; 5 Joint BioEnergy Institute, Emeryville, CA 94608, USA; 6 Department of Chemical Engineering, University of California, Berkeley, CA 94720, USA

## Abstract

**Background:**

Standard biological parts, such as BioBricks™ parts, provide the foundation for a new engineering discipline that enables the design and construction of synthetic biological systems with a variety of applications in bioenergy, new materials, therapeutics, and environmental remediation. Although the original BioBricks™ assembly standard has found widespread use, it has several shortcomings that limit its range of potential applications. In particular, the system is not suitable for the construction of protein fusions due to an unfavorable scar sequence that encodes an in-frame stop codon.

**Results:**

Here, we present a similar but new composition standard, called BglBricks, that addresses the scar translation issue associated with the original standard. The new system employs BglII and BamHI restriction enzymes, robust cutters with an extensive history of use, and results in a 6-nucleotide scar sequence encoding glycine-serine, an innocuous peptide linker in most protein fusion applications. We demonstrate the utility of the new standard in three distinct applications, including the construction of constitutively active gene expression devices with a wide range of expression profiles, the construction of chimeric, multi-domain protein fusions, and the targeted integration of functional DNA sequences into specific loci of the *E. coli *genome.

**Conclusions:**

The BglBrick standard provides a new, more flexible platform from which to generate standard biological parts and automate DNA assembly. Work on BglBrick assembly reactions, as well as on the development of automation and bioinformatics tools, is currently underway. These tools will provide a foundation from which to transform genetic engineering from a technically intensive art into a purely design-based discipline.

## Background

Synthetic biology takes a ground-up approach to genetically engineer cellular systems capable of the sophisticated sensing, information processing, and actuation exhibited by natural systems. While it is important to build increasingly complex systems when necessary, the goal is to do so using tools and methodologies that streamline biology and make it easier to engineer. At the center of this approach lies the need to impart novel biological function(s) by systematically introducing new designed DNA sequences into living cells. The two main challenges in this endeavor are: first, knowing how to design sequences that impart a particular function; and second, how to construct the DNA encoding such function in a form that can be readily introduced into the cell. Standard assembly parts, such as BioBricks™ [1], provide a paradigm that addresses both of these problems by recognizing that functional units of DNA sequence are frequently reused in a variety of projects. These units, which include promoters, ribosome binding sites, protein coding sequences, among others, represent non-reducible elements of genetic composition, and as such, are considered "basic parts." The sequences of each part are stored within databases, such as the Registry of Standard Biological Parts [2], while the physical DNAs are housed in part collections. Each basic part is a genetic element that has been refined to comply with any of several publicly-available standards and is not associated with one particular assembly strategy. Nevertheless, because each part is standardized to conform to a defined set of rules, a single standard assembly reaction can be used to concatenate basic parts. Furthermore, because only one standard assembly reaction is required to iteratively combine any two parts, we can assemble multi-part devices and characterize the rules of functional composition for each part in the context of other parts [3,4]. Thus, by standardizing the basic part junction sequence, the task of defining contextual rules for part function is significantly narrowed. We envision that a robust standardized assembly process will enable the development of low-cost, high-throughput, automated assembly facilities, and ultimately, the outsourcing of entire DNA fabrication processes at a reasonable price.

The BioBricks™ standard described by Knight and coworkers was the first implementation of a strategy for defining composition rules that allow the assembly of standard biological parts using a single assembly chemistry [1]. The assembly method employs iterative restriction enzyme digestion and ligation reactions to assemble small basic parts into larger composite parts. Basic parts are flanked by XbaI and SpeI restriction sites on their 5' and 3' ends, respectively. Digestion with these enzymes generates compatible cohesive ends that can be ligated back together head-to-tail. The ligation of two parts generates a scar sequence between the parts that contains neither of the original sites, and thus, it is unaffected by subsequent digestion with either XbaI or SpeI. The resulting product is a new composite part with the same assembly characteristics as the two parent parts. It is still flanked by unique XbaI and SpeI restriction sites on its 5' and 3' ends, respectively, and hence, the iterative assembly of larger and larger composite parts becomes possible. Over 2,000 basic parts that conform to this standard have been described, and they have been used in the construction of a wide range of genetic circuits and biosynthetic devices [5-8].

Since the inception of the first assembly standard, several others have been proposed and/or developed to describe functional composition and/or physical assembly. In fact, this field is currently undergoing robust activity, and the number of assembly standards is expanding rapidly. At present, the BioBricks Foundation (BBF) has implemented an organizational framework, known as a BBF RFC (request for comments) process, to help define, evaluate, and propose new standards in the field [9]. As an example, we refer the reader to BBF RFC 29 which describes the major assembly standards proposed to date and suggests an organized naming process for future standards [10]. One issue with the original BioBricks™ standard, and addressed herein, is the ability to compose protein-fusion parts encoding elements such as peptide tags and single domains of polypeptides for protein engineering applications.

Modular protein engineering is an emerging area of synthetic biology. Several studies have shown the power of building sophisticated protein machines by assembling multiple modular domains into a variety of larger polypeptide sequences [6,11-14]. Unfortunately, the original BioBricks™ assembly scheme (BBF RFC 10) is not suitable for building chimeric proteins because the 8-nucleotide scar sequence that remains between parts after they have been joined together is incompatible with protein fusions for two reasons: first, the scar sequence (TACTAGAG) encodes tyrosine followed by a stop codon; and second, an 8 nucleotide scar inserts a frame shift between the two coding sequences. Theoretically, this problem could be addressed by elongating the scar sequence to contain a number of nucleotides that is a multiple of 3, such that the stop codon is no longer in frame and the frame shift is eliminated. In the mean time, a number of improvements to the original BioBricks™ standard, along with completely alternative assembly strategies and standards, have been proposed and/or developed. The Biofusion standard (BBF RFC 23), for example, modifies the original BioBricks™ standard to create a smaller, 6 nucleotide scar sequence (ACTAGA) that encodes threonine-arginine, and thus, eliminates both reading frame shifts and encoded stop codons [15]. Unfortunately, the AGA codon encoding arginine is a rare codon in *E. coli, *and furthermore, the XbaI site can be blocked by *dam *methylation when flanked by certain sequences [15]. The Fusion Parts standard (BBF RFC 25), developed by the Freiburg 2007 iGEM team, is another extension of the BioBricks™ standard that seeks to alleviate some of the disadvantages of the Biofusion format. Here, AgeI and NgoMIV restriction sites are used to generate a 6 nucleotide scar sequence (ACCGGC) encoding threonine-glycine. Finally, the BioBricks++ standard is a scarless assembly standard that uses two steps for assembly [16]. It uses type IIs restriction enzymes to recognize sites flanking the part and digest at the boundary of the part. The cohesive ends are then blunted prior to scarless ligation, which is the ultimate goal of standard assembly strategies. Unfortunately, robust reactions necessary to implement such a method have yet to be identified. All of these standards, along with a complete list of their advantages and disadvantages, are described further at the BBF's Standards and Formats page [17].

Here we describe a new robust, yet flexible, standard for composing biological parts called BglBricks [18]. The new standard addresses several of the key problems associated with the original BioBricks™ standard, and furthermore, it provides a foundation for developing automated assembly platforms. The BglBrick standard supports assembly with the BglII and BamHI restriction enzymes flanking the 5' and 3' ends of basic parts, respectively. These enzymes possess several advantages over the ones used in previous standards: first, they have an extensive history of use, which ensures their reliability; second, they cut with high efficiency; third, they are unaffected by overlapping *dam *or *dcm *methylation; finally, they result in a 6-nucleotide scar sequence (GGATCT) encoding glycine-serine, a sequence demonstrated to be innocuous in most protein fusion applications in a variety of host systems, including *E. coli, *yeast, and humans [19-21]. In the following sections, we showcase uses of the BglBrick standard in 3 diverse applications. These include the construction of constitutively active gene expression devices that elicit a wide range of expression profiles; the construction of chimeric, multi-domain fusion protein expression devices; and finally, the targeted integration of parts and devices into specific loci of the *E. coli *genome.

## Results

### Definition of the BglBrick standard

At its core, the BglBrick standard is a composition standard that enables idempotent assembly schemes using BglII and BamHI restriction enzymes for assembly of biological parts. As defined herein, the BglBrick standard deals with the theoretical composition of DNAs, but not their physical assembly. While it is designed as a minimal standard that enables vector and part compatibility and the precise description of the products of restriction enzyme-based manipulations, it imposes no further explicit rules on composition. Here, only the rules needed to support part and vector description, interconversion with EcoRI and BamHI, and concatenation with XhoI, BamHI, and BglII are formally defined (Table 1). Thus, the omission of extensive assembly details is intentional given that BglBrick parts can be assembled using a variety of different methods, including PCR-based assembly. A BglBrick part is defined as a DNA sequence flanked by "GATCT" on the 5' end and by "G" on the 3' end, and lacking internal BamHI, BglII, EcoRI, and XhoI restriction sites. A BglBrick vector is defined as a DNA sequence flanked by "GATCC" on its 5' end and by "A" on its 3' end. A BglBrick plasmid is defined as a BglBrick vector plus a BglBrick part, where the part can be either basic or composite. The nomenclature for a BglBrick plasmid is "vectorName-partName," and its sequence is the concatenation of its vector and part(s) sequences. Each vector or part sequence carries a unique name corresponding to its exact sequence. Derivatives of a BglBrick vector or part differing by even a single nucleotide carry a unique name. Most (but not all) BglBrick plasmids encode an EcoRI-BglII-BglBrick part-BamHI-XhoI cassette. A BglBrick entry vector is a special type of vector used to harbor basic parts and support their assembly, and is defined as a sequence containing a unique EcoRI site, no BamHI or BglII sites, and at most one XhoI site 5' to the EcoRI site. The EcoRI site is used to facilitate part transfers between different vectors. It can also be used for "prefix" insertion assembly reactions (the prefix region is the vector fragment between EcoRI and BglII). Similarly, if present, the XhoI site can be used to facilitate "suffix" insertion assembly reactions (the suffix region is the vector fragment between BamHI and XhoI). Given the core definition of the BglBrick standard as a minimal assembly standard that guarantees compatibility but imposes no explicit rules on composition, start and stop codons are not part of the definition. Similarly, the core definition places no constraints on the location of other restriction sites, origins of replication, and antibiotic selection markers, or on the length and sequence of the "prefix" and "suffix" regions. Such further constraints, considered sub-standards of the BglBrick format, are dynamic community-defined rules collected and described by the BBF RFC process [9]. These sub-standards may include rules for the location of the start codon of a coding sequence, locations of other restriction sites, vector sets for specific standard assembly protocols, etc.

**Table 1:** Core definitions of the BglBrick standard

BglBrick plasmid	A BglBrick vector plus a BglBrick part, where the part can be either basic or composite. The nomenclature for a BglBrick plasmid is "vectorName-partName" and its sequence is the concatenation of its vector and part(s) sequences.
BglBrick part	A DNA sequence flanked by "GATCT" on the 5' end and by "G" on the 3' end, and lacking BamHI, BglII, EcoRI, and XhoI restriction sites.

BglBrick vector	A DNA sequence flanked by "GATCC" on its 5' end and by "A" on its 3' end.

BglBrick entry vector	A special type of vector sequence containing a unique EcoRI site, no BamHI or BglII restriction sites, and at most one XhoI site 5' to the EcoRI site.

### Standard assembly

**Figure 1:**
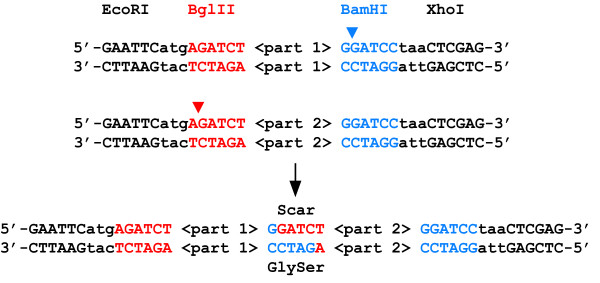
**Standard assembly of BglBrick parts**. Unique BglII (in red) and BamHI (in blue) restriction sites flank BglBrick basic parts on their 5' and 3' ends, respectively. EcoRI and XhoI restriction sites employed in various protocols for part assembly are also shown. Cleavage of each DNA with the appropriate enzyme (color-coded arrowheads) generates compatible cohesive ends. These can be connected head-to-tail by ligation (black arrow) to generate composite parts separated by a 6 nucleotide scar sequence (ggatct). When translated in frame, the scar sequence between parts encodes glycine-serine, a peptide linker innocuous for most protein fusion applications.

Prefix insertion is one strategy for standard assembly of BglBrick parts facilitated by plasmids containing a BglBrick entry vector. Such plasmids encode unique BglII and BamHI restriction sites flanking the 5' and 3' ends, respectively, of the parts to be joined (Figure 1). Briefly, to join two basic parts A and B, in that order, plasmid containing part A is digested with BamHI (which cuts 3' of the part) and EcoRI (which cuts the vector), while plasmid containing part B is digested with BglII (which cuts 5' of the part) and EcoRI. The two basic parts are connected head-to-tail by ligation to generate a composite part, which itself is flanked by unique BglII and BamHI sites on its 5' and 3' ends, respectively. Within the new composite part, parts A and B are separated by a 6-nucleotide scar sequence. When translated in frame, the scar sequence encodes glycine-serine, an innocuous peptide linker for protein fusions. To date, over 1,000 distinct BglBrick plasmids have been constructed. In the following sections, we showcase use of the standard in 3 diverse applications. These include the construction of constitutively active gene expression devices that elicit a wide range of expression profiles; the targeted integration of parts and devices into specific loci of the *E. coli *genome; and the construction of chimeric, multi-domain protein expression devices.

### Assembly of protein expression devices with ranging expression profiles

**Figure 2:**
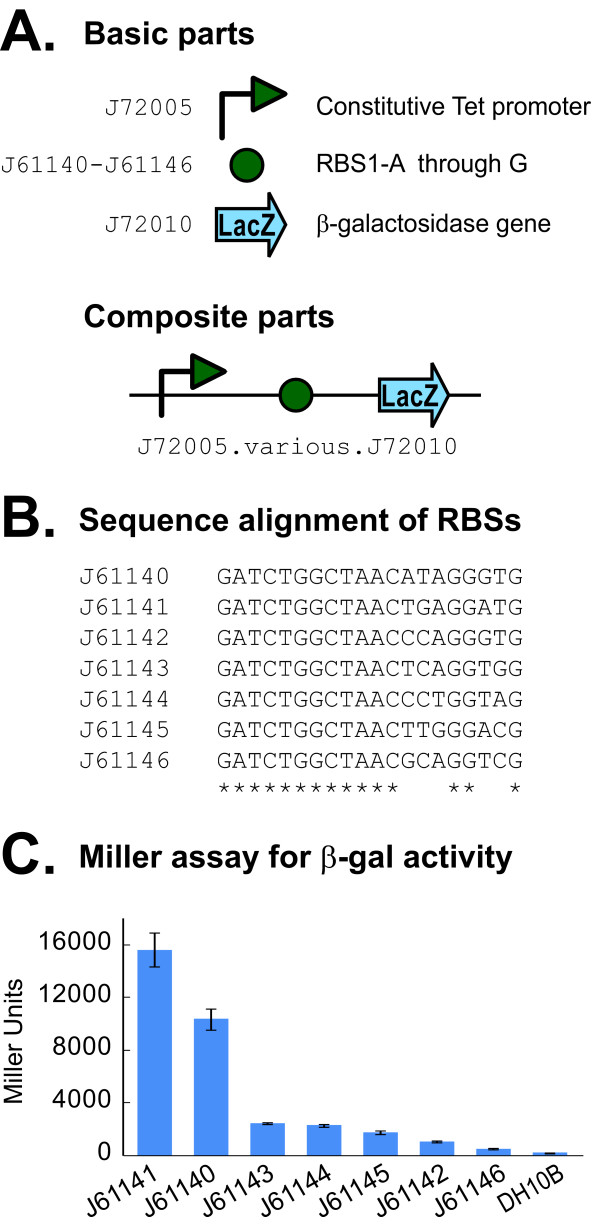
**Assembly of *lacZ *expression devices**. (A) Standard assembly of BglBrick basic parts were used to generate a series of constitutive *lacZ *expression devices. Each composite part consisted of a different ribosome binding site part located between promoter and *lacZ *coding sequence parts. (B) Sequence alignment of RBS variants. Asterisks indicate sequence identity. (C) The β-galactosidase activity of each device (labeled for RBS parts J61140-J61146), along with a negative control lacking a device (DH10B cells alone), is shown. For each, the average of 5 individual replicates is shown. A wide range of activities were observed, indicating the ability to tune protein expression levels using BglBrick RBS part libraries.

One of the recurring goals of synthetic biology is to predictably and reliably identify DNA sequences that confer a specific biological property at a precise level of activity. A generalized approach to this goal is to encapsulate quantitative information about the function of specific parts. To begin to address this, we tested a library of ribosome binding sites (RBSs) for their ability to confer varied levels of translation efficiency in the context of the BglBrick format (Figure 2). We first created a library of RBS parts in a BglBrick plasmid by saturation mutagenesis. Of the mutants obtained, we selected several variants that spanned the range of RBS activities observed within the library for use as basic parts. Using standard assembly, we constructed a series of composite parts consisting of a constitutively active Tet promoter, one RBS variant, and a *lacZ *coding sequence. The β-galactosidase activity of each variant was determined by Miller assay and showed a 100-fold range of activity over the conditions tested. These experiments demonstrate that BglBrick assembly can be used to create a series of functional protein expression devices exhibiting a wide variety of expression profiles.

### Assembly of multi-domain fusion protein devices

One of the main advantages of the BglBrick standard is that the 6 nucleotide scar produced after assembly of two adjacent parts encodes a glycine-serine flexible linker when translated in-frame. To demonstrate that the BglBrick standard can be used to assemble functional fusion protein devices composed of several ORF parts, we constructed devices encoding multiple domains, including several repeats of protein-protein interaction motifs. The devices were engineered to encode "bait" proteins, and tested for their ability to pull down "prey" using *in-vitro *pull down assays. To make the bait devices, we first assembled composite parts containing either 0, 1, or 4 repeats of an SH3 interaction motif containing an N-terminal glycine-serine linker that were separated by the BglBrick scar sequence, which added an additional glycine-serine repeat. We also used the BglBrick standard to tether these composite parts to the C-terminus of the enzyme HMG-CoA synthase (HMGS). Additionally, HMGS was N-terminally tagged with GST to aid in protein purification. As prey, we used HMG CoA reductase (HMGR), N-terminally tagged with an SH3 interaction domain [6]. In these experiments, HMGS and HMGR were used as "carriers" of the bait SH3 peptide ligand repeat and the prey SH3 domain, and thus, should be considered inert for the desired binding function.

Restriction enzyme-based assembly is a particularly attractive strategy for the construction of devices with repetitive elements, which can be problematic for two main reasons. First, devices containing repetitive sequences are difficult to create using PCR-based methods because misaligning and erroneous annealing of primers often produces shorter than desired products. Second, sequences containing repetitive elements can be targets for recombination, which can often lead to deleted and/or rearranged devices. In this application, we lowered the risk of homologous recombination occurring in the cell by creating three degenerate basic parts in the BglBrick format, all encoding SH3 ligand motifs, but each containing a distinct sequence. Combinations of these three degenerate parts were used to assemble repetitive composite parts by standard assembly. Although we have not observed occurrences of recombination with devices containing up to four identical repeats, this can be problematic for assembling devices with larger numbers of identical repeats (JED unpublished data).

**Figure 3:**
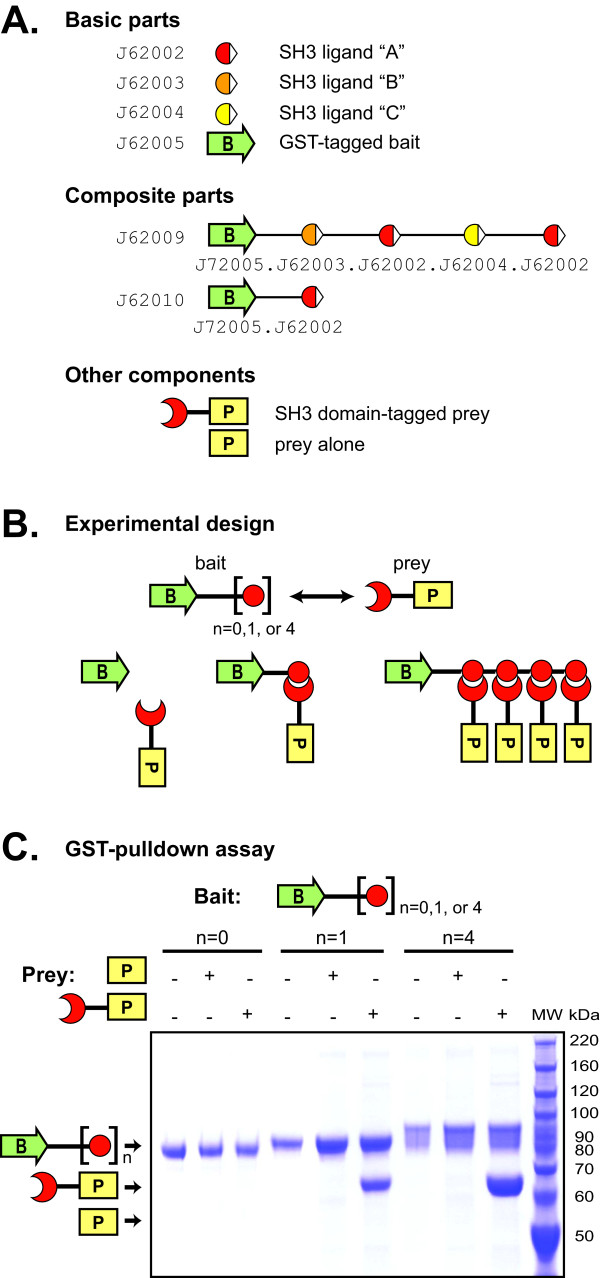
**Assembly of multi-domain fusion protein devices**. (A) Basic parts containing protein-protein interaction ligands were built using three independent, degenerate sequences encoding an N-terminal glycine-serine linker and an SH3 interaction peptide. Composite parts were created by standard assembly of these basic parts. (B) Experimental design for testing interactions between bait and prey parts. The number of prey molecules pulled-down by bait should be dependent on the number of SH3 peptide ligands. (C) A GST-pulldown experiment was conducted and the proteins separated via SDS-PAGE and then imaged by Coomassie staining. No visible interaction was observed when either the bait lacked any SH3 peptide ligand or the prey lacked an SH3 domain. Bait containing composite part J62008 with four SH3 peptide ligands, despite visible proteolytic degradation, was sufficient to pull down increased amounts of prey compared to bait containing the single SH3 ligand part J62002.

To show the functionality of our multi-domain fusion protein devices, each bait was tested for its ability to form a synthetic complex with prey in an *in-vitro *GST-pulldown assay (Figure 3). Baits with and without SH3 peptide ligand motif(s) ran at the expected mobility. Protein-protein interactions and complex formation were only observed in the presence of bait containing SH3 peptide ligand motif(s) and prey containing an SH3 interaction domain. Furthermore, baits containing 4 protein interactions domains were able to pull down more prey than baits containing a single interaction domain, indicating as expected, that the amount of prey pulled down correlates with the number of SH3 peptide ligand motifs present on the bait. Conversely, no complex formation was observed when bait and prey lacking protein interaction domains were used in the pull-down. These data agree well with data obtained for similar constructs made using a non-BioBricks restriction enzyme strategy [6]. Furthermore, they demonstrate that BglBrick parts can be used to create functional multi-domain fusion protein devices containing repetitive elements and that the BglBrick scar sequence can be inserted between expressed protein domains without significantly affecting function.

### Targeted integration of BglBrick devices into the E. coli genome

Another recurring goal in synthetic biology is to engineer microbial strains that express introduced genes in a stable and predictable manner, either in single copies or as part of complex genetic circuits. To achieve this level of regulation, we need tools that facilitate the targeted integration of DNA sequences into specific regions of the bacterial chromosome (for a minireview of site-specific recombination see [22]). Wanner and coworkers have described a genomic integration system, based on CRIM plasmids (for conditional-replication, integration, and modular), that enables single copy integration of multiple plasmids into several defined loci of one *E. coli *genome [23]. The CRIM plasmids carry an R6K conditional origin of replication, a selectable antibiotic resistance marker, and a phage attachment site (*attP*). By supplying a helper plasmid encoding integrase (Int), a CRIM plasmid can be site-specifically recombined into its corresponding bacterial attachment site (*attB*). In the absence of Int, CRIM plasmids can be maintained extra-chromosomally provided that the host strain expresses *pir*, the gene required for replication of R6K origins.

The CRIM system is a versatile tool because its modularity allows the use of alternative *attP *and *attB *sites (for phages λ, HK022, Φ80, P21, and P22, all of which target distinct sites of the *E. coli *genome), various antibiotic resistance markers, as well as several polylinkers and inducible promoters. We have made three modifications to render the system more robust and compatible with the BglBrick standard. First, we have facilitated CRIM plasmid integration by adding the *pir *gene needed for replication of R6K origins to the helper plasmid. This ensures that CRIM plasmids will be replicated prior to integration, which, in turn, increases the chances of a successful integration event. Second, we have refactored the essential elements of CRIM plasmids into distinct BglBrick basic parts from which new CRIM plasmids can be assembled. Finally, we have made marker-less integration variants of CRIM plasmids, such that, if desired, antibiotic resistance markers can be removed from the cell after the integration process is complete. This is desirable when an application requires the reuse of an antibiotic resistance marker in the engineered strain, or for high-volume or environmental release applications where retention of antibiotic resistance is potentially unsafe.

**Figure 4:**
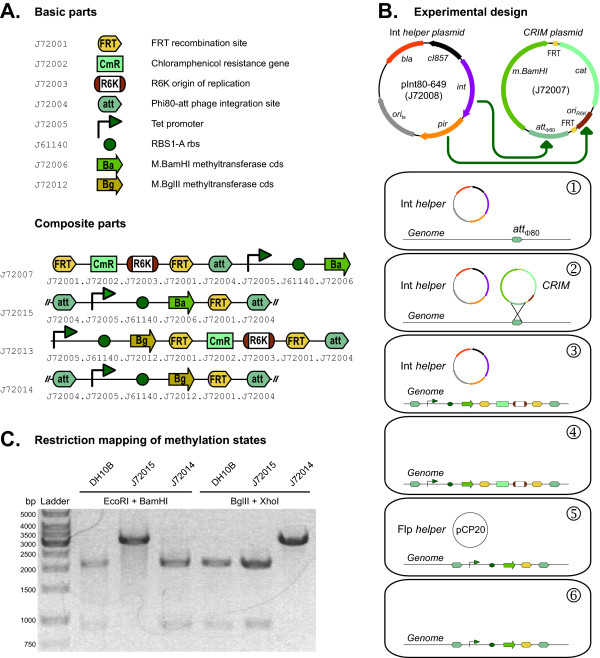
**Targeted integration of BglBrick parts into the *E. coli *genome**. (A) A variety of basic parts were used to create two methyltransferase expression devices targeting BamHI and BglII restriction sites (parts J72007 and J72013, respectively). Each device was recombined into the genome of strain MC1061 by Φ80 *att *site integration, resulting in BamHI- and BglII-methylating strains (parts J72015 and J72014, respectively). (B) Sample experimental design for genomic integration of CRIM plasmids. Circuit components are color-coded and graphically represented relative to (A) and the top of (B) for easy identification. (1) Host strain MC1061 with unmodified genomic Φ80 *att *sites is transformed with temperature sensitive helper plasmid pInt80-649 (part J2008) and selected on ampicillin plates. (2) Cells are re-transformed with CRIM plasmid (part J72007), which replicates as a high-copy R6K plasmid employing the *pir *gene provided by the helper plasmid. (3) The CRIM plasmid inserts into the genome by recombination with the Φ80 *att *site employing the *int *gene from the helper plasmid. (4) Helper plasmid is cured by growth at 43°C. (5) Helper plasmid pCP20 encoding Flp recombinase is introduced by transformation and the R6K origin and chloramphenicol genes are excised from the genome by recombination of FRT sites. (6) Helper plasmid is cured by growth at 43°C, resulting in the final product containing a genomically-integrated BglBrick part and a single FRT site. (C) Restriction mapping of plasmid J61148-J72011 isolated from BglII- and BamHI- methylating strains (parts J72015 and J72014, respectively) or DH10B (control) confirms the appropriate protection.

CRIM plasmids contain three essential elements: a conditional origin of replication, a selectable marker, and a phage attachment site (*attP*). Additional elements in each CRIM plasmid will vary according to its intended purpose. To make functional CRIM plasmids via standard assembly, we first had to construct basic parts for both the essential and additional elements used in this application (Figure 4a). For the CRIM elements, we maintained R6K as the conditional origin of replication, we used chloramphenicol as the selectable marker, and opted for the Φ80 phage attachment site because the Φ80 CRIM plasmid was shown to undergo genome integration most efficiently [23]. Though we highlight Φ80 parts here, we also constructed P21 integration parts (not shown). Additional elements used in this application include basic parts containing a constitutively active Tet promoter and an RBS described in other sections of this report; two parts encoding different methyltransferases; and one part containing an FRT recombination site. By flanking sequences with FRT recombination sites, these can be later targeted for elimination by Flp-mediated recombination [24]. We have used this tool to remove antibiotic resistance markers from the *E. coli *genome following initial integration of CRIM plasmids.

To functionally test the integration parts, we made independent strains of *E. coli *that methylate either BglII or BamHI restriction sites. Such strains will be useful for the development of next-generation BglBrick standard assembly chemistries. First we used BglBrick basic parts and standard assembly to build CRIM plasmids carrying methylation devices (Figure 4a). In addition to the essential CRIM elements, the plasmids contained a BglII or BamHI methyltransferase expression cassette composed of a Tet promoter, an RBS, and the corresponding methyltransferase ORF. Also using standard assembly, we flanked the R6K origin and the antibiotic resistance marker of each CRIM plasmid with FRT recombination sites to target them for removal after genome integration. For the experiment, plasmids containing methylation devices were introduced into MC1061 cells previously transformed with a helper plasmid carrying *pir *and the corresponding Φ80 *int *gene (Figure 4b). Following integration into the MC1061 genome, selection, curing of any remaining extra-chromosomal DNA, and subsequent removal of the R6K and chloramphenicol resistance gene by Flp-mediated recombination, we obtained new strains of *E. coli *that are capable of methylating BglII and BamHI sites on plasmids replicating within them. To confirm the presence and functionality of this genomically-encoded activity, we transformed each strain with a BglBrick plasmid (J61148-J72011) that contains a 910-bp BglBrick part flanked by EcoRI and BglII on the 5' end, and by BamHI and XhoI on the 3' end. Following methylation, we purified the plasmid and analyzed its methylation state by restriction enzyme mapping (Figure 4c). As expected, digestion of control unmethylated DNA with either BglII and XhoI, or BamHI and EcoRI, liberates the 910-bp BglBrick part-containing fragment carried in the test plasmid. In contrast, digestion of the same plasmid purified from the BglII or BamHI methylation strains were protected from BglII or BamHI digestion, and instead, were only linearized by XhoI or EcoRI. Collectively, these experiments demonstrate that BglBrick parts are viable tools for the creation of CRIM plasmids that can be used to specifically target DNAs carrying functional devices into defined loci of the *E. coli *genome.

## Discussion

The primary bottleneck in synthetic biology research today is the construction of physical DNAs, a process that is often expensive, time consuming, and riddled with cloning difficulties associated with the uniqueness of each sequence. A variety of strategies are available to tackle DNA construction projects. At one end of the spectrum, there are *de novo *synthesis techniques to build DNA sequences starting from single bases, while at the other end, there are assembly schemes that begin with physical collections of short DNA sequences and fuse them head-to-tail to generate larger constructs. Alongside more traditional restriction enzyme-based assembly methods, multiple techniques within this spectrum are often used in combination. To name a few, Gene Splicing by Overlap Extension (gene SOEing), is a sequence-independent, PCR-based method, that enables site-directed mutagenesis and/or recombination of DNA sequences without relying on restriction enzyme sites [25,26]. Sequence and Ligation Independent Cloning (SLIC) is another PCR-based method that allows the assembly of multiple DNA fragments in a single reaction using *in-vitro *homologous recombination and single-strand annealing [27]. More recently, there have been reports of a single-reaction DNA shuffling method based on type IIs restriction enzymes, known as Golden Gate Shuffling [28], as well as a single-reaction isothermal method for the assembly of multiple overlapping DNA molecules [29]. Similarly, Tsuge and coworkers have reported a cloning technique to iteratively construct large sequences of DNA, on the scale of small genomes, and to transfer them into host cells [30]. Despite these and other encouraging advances, every methodology has unique advantages and flaws, and thus, there is still no single DNA fabrication protocol that addresses all assembly needs. For example, even though prices for *de novo *gene synthesis continue to drop, in practice the technique remains prohibitively expensive, especially for high-throughput applications. As a result, most synthetic biology labs routinely employ assembly strategies even when the core elements of their designs are made using *de novo *gene synthesis. Similarly, though assembly reactions are inexpensive in comparison to *de novo *gene synthesis, they are complicated by the uniqueness of each assembly strategy, by errors incurred during PCR-based methods, and by the need for extensive manual operations, including pipetting, cutting and pasting reactions, gel purifications, transformations, etc. As they stand, the existing *de novo *gene synthesis and assembly strategies are complementary technologies that tackle different scales of DNA fabrication.

Strategies for the standard assembly of biological parts emerge from the recognition that the power of standardized parts lies in their ability to separate function from assembly, and furthermore, to sustain the development of a single robust reaction chemistry and protocols suitable for all assembly applications. Because each part has a standardized interface for assembly, each is guaranteed to cut in the right place and assemble in the same way as other parts conforming to the same standard. In idempotent assembly schemes, such as the BioBricks™ paradigm, the child of assembling two basic parts is itself amenable to assembly using the same assembly reaction. As a result, any number and order of basic parts can be assembled into larger DNA cassettes through iterated cycles of identical operations. Thus, the problem of composition can be pursued without consideration for how the DNAs are assembled. Similarly, the reliability, cost-effectiveness, throughput, and automation of assembly reactions can be developed without consideration of composition. With the increasing complexity of engineered biological systems, there is great need for robust standard assembly methods that are able to rapidly translate designed compositions into physical entities while minimizing financial and time costs.

The first step in implementing an idempotent standard assembly strategy requires the definition of technical standards for parts, such that all parts are amenable to the same assembly reaction. Here we have described a new flexible standard for composing biological parts called BglBricks. In developing the BglBrick standard, we recognized that the utility of any assembly strategy is principally based on two factors: first, the intrinsic robustness of the restriction enzymes involved in the assembly process; and second, the desirability of the scar sequence that remains between parts after they have been joined together. We have addressed these points by selecting enzymes with an extensive history of use, demonstrated reliability, high cutting efficiency, and resulting in an innocuous scar sequence that enables protein fusion applications in a variety of common host systems. Consequently, the resulting BglBrick standard represents an improvement over the original BioBricks™ assembly strategy, whose primary shortcoming is an inability to support the construction of protein fusions. Finally, we have showcased use of the BglBrick standard in 3 diverse applications and demonstrated that it can be used for most DNA assembly scenarios encountered in synthetic biology experiments. To date, thousands of distinct biological parts have been constructed using the BglBrick standard, and methods for assembly continue to be actively developed.

## Conclusions

The BglBrick standard supports robust reaction chemistries that can be applied for most DNA assembly applications, including those required for the construction of multi-domain protein fusions. In so doing, the BglBrick standard not only separates the laborious problem of DNA assembly from its functional design, but it also sets the foundation needed for future automation and scaling of these processes. Toward this goal we are developing a variety of approaches, technologies and reagents that will eventually enable standard assembly of multi-component genetic systems *in vivo *using only automated liquid handling operations. We believe these tools will provide a foundation from which to transform and simplify genetic engineering.

## Methods

### Reagents and strains

DNA-modifying enzymes were purchased from New England Biolabs.

Oligonucleotides were synthesized by Sigma-Genosys or IDT and used unpurified. PCR was performed with the Roche Expand High Fidelity PCR kit. All reactions were performed according to the manufacturer's instructions. DNAs were transformed into bacteria by heat shock using KCM chemically competent cells. Unless otherwise noted, all manipulations were performed in *E. coli *strain DH10B grown at 37°C in LB liquid medium or agar plates, supplemented with antibiotics at 100 μg/mL for ampicillin, and 25 μg/mL for all others.

### Construction of basic parts

Detailed descriptions of the construction of each BglBrick basic part-encoding plasmid are provided on individual part pages available at the Registry of Standard Biological Parts [2] and may be located by their part number. Using various conventional molecular biology techniques, DNA cassettes encoding basic parts flanked by BglII and BamHI restriction sites at their 5' and 3' ends, respectively, were inserted into entry vector-containing plasmid pBca9145 (part J61148), which contains a ColE1 origin of replication, an ampicillin resistance gene, and the BglBrick polylinker. As an example, the plasmid resulting from introduction of the J72005 Ptet promoter part into pBca9145 is J61148-J72005.

### Standard assembly of basic parts

Although multiple specific protocols for BglBrick standard assembly are currently in development, the general strategy described here can be referred to as suffix insertion. Briefly, to join two plasmids containing parts A and B, in that order, plasmid containing part A is digested with BamHI (which cuts 3' of the part) and XhoI (which cuts the vector). Similarly, plasmid containing part B is cut with BglII (which cuts 5' of the part) and XhoI. DNA fragments are separated by electrophoresis, parts of interest are gel purified, ligated, and transformed into *E. coli *for characterization and maintenance.

### Construction and screening of a ribosome binding site library

Plasmid pSB1A2-J23100 (part J23100), which encodes only a constitutive promoter, was used as a template to PCR amplify a library of RBSs downstream of the promoter by PCR, using oligonucleotides CA998 (5'-GTATCACGAGGCAGAATTTCAG-3'), which targets the vector backbone, and CA1106R (5'-CTTGCGGATCCNNCCNNNGTTAGCCAGATCTAGCTAGCACTGTACCTAGGACTG-3'), which includes 5 degenerate bases that target the intended Shine-Delgarno sequence. PCR products were digested with EcoRI and BamHI, and ligated into the EcoRI and BamHI sites of reporter plasmid pBca1101 (part J72009). Reporter plasmid pBca1101 lacks a promoter driving expression of a downstream RFP gene, so ligation of the degenerate PCR products creates devices consisting of a constitutive promoter, RBS and RFP gene. From the transformants obtained, 96 individual clones were assayed for RFP expression in deep-well microtiter plates using a Tecan Safire 2 fluorescence plate reader. Several distinct variants, named RBS1-A through RBS1-G (parts J61140-J61146), that span the range of expression activities observed within the library were selected for further characterization and use as basic parts in standard assembly reactions.

### Analysis of lacZ expression devices

Composite *lacZ *expression devices consisting of a constitutively active Tet promoter (part J72005), one of the variants identified in the RBS library screen, and a β-galactosidase coding sequence (part J72010), were generated via BglBrick standard assembly reactions with vector pBca9145 (part J61148). For each *lacZ *expression variant tested, 5 individual colonies were assayed for β-galactosidase activity. Cells were grown to saturation in LB medium supplemented with ampicillin, diluted 1:50 in fresh medium, and then grown to OD_600 _= 0.5. Activity was determined according to a modified Miller assay [31].

### Construction of multi-domain fusion protein devices

Composite parts encoding multiple SH3 peptide ligands were constructed from three, degenerate, single-ligand basic parts (parts J62002, J62003, and J62004) by standard assembly reactions. To limit the probability of recombination due to repetitive sequences, each ligand basic part was engineered with degenerate codon usage. Each ligand part also included a degenerate sequence encoding an N-terminal glycine-serine linker predicted to be unstructured and flexible that was codon-optimized for expression in *E. coli*. From these individual degenerate parts, it was possible to assemble any combination of SH3 ligands separated by flexible linkers that include the scar sequence. As an example, part J62008, which contains four SH3 peptide ligand domains, was built by joining two composite parts, each with two ligand domains (parts J62006 and J62007). For this application, it was necessary to assemble the SH3 peptide ligand cassettes into "bait" expressing devices that could pull-down "prey" molecules. Theoretically, bait can be any protein fused to these SH3 ligands, while prey can be any protein fused to a corresponding SH3 domain. Bait-expressing plasmids were constructed by digesting HMG-CoA synthase-encoding plasmids (HMGS) (part J62005) with BglII and XhoI, and ligating into the T7-driven GST-fusion expression vector pGEX 4T-1 (Pharmacia). The resulting vector was digested with BamHI and XhoI and used as a backbone for inserting the BglBrick assembled interaction ligands digested with BglII and XhoI. Consequently, bait was expressed as an N-terminal GST-tagged HMGS with a variable number of SH3 ligands fused to its C-terminus. Prey was constructed using splicing by overlapping extension (SOE-PCR) to fuse the SH3 domain to the N-terminus of HMG-CoA reductase (HMGR). In these experiments, HMGS and HMGR were used as "carriers" of the bait SH3 peptide ligand repeat and the prey SH3 domain, and thus, should be considered inert for the desired binding function.

### Fusion protein over-expression, purification, GST pull-down assays

*E. coli *strain BLR-DE was used to express all baits and preys used in these pull-down assays (GST-HMGS-baits with and without SH3 peptide ligands, and HMGR-preys with and without SH3 domain). Briefly, cells were grown overnight at 37°C in 5 mL LB supplemented with ampicillin. The next morning, cultures were diluted to an OD_600 _= 0.05 into 500 mL of LB supplemented with ampicillin. Cultures were further grown to an OD_600 _= 0.5-0.7 and subsequently induced with 0.5 mM IPTG for 5 hours. Cells were isolated by centrifugation, resuspended in 35 mL of PBS buffer, and lysed by sonication. A fast centrifugation step was done to clear the lysate. The soluble supernatant was split into 1.5 mL aliquots, flash frozen in liquid nitrogen, and stored at -80°C until further use. In preparation for the pull-downs, GST-tagged baits were affinity purified away from cell supernatants using glutathione agarose beads (Sigma). Briefly, lysate aliquots were slowly thawed on ice and incubated with beads pre-washed in PBS buffer at 4°C for 30 min. They were then isolated by centrifugation using a desktop Galaxy minicentrifuge (VWR) for approximately 10 sec., and washed four times with fresh PBS buffer at 4°C. For the pull-down, the bead-bound, purified bait was mixed with unpurified, cleared prey lysate and incubated at 4°C for 30 min. Complexes were isolated by centrifugation as above, and washed four times with fresh PBS buffer supplemented with 0.05% Tween-20 at 4°C. The samples were then resolved on 4-12% Bis-Tris NuPAGE gels (Invitrogen) and visualized by Coomassie stain.

### Integration of CRIM plasmids into the E. coli genome

Detailed descriptions of the construction of helper plasmids, as well as basic and composite parts used in this section, are provided on the individual part pages at the Registry of Standard Biological Parts [2]. After assembly of full-length CRIM composite parts, such as J72007 (used as example in Figure 4c), the original ColE1 origin of replication and ampicillin resistance marker from the initial vector backbone were excised by digestion with BglII and BamHI. The desired CRIM composite part was re-circularized by ligation and introduced into *E. coli *strain Ec100D::pir116 (Epicentre) for maintenance as high-copy plasmids on chloramphenicol-supplemented medium. For genomic integration, the target *E. coli *strain MC1061 was first transformed with helper plasmid pInt80-649 (part J72008), which contains an ampicillin marker, a temperature-sensitive origin of replication for maintenance, the *int *gene for Φ80 *att*-site integration, and a *pir116 *gene. Transformants were grown on ampicillin plates under permissive conditions at 30°C. The resulting cells were made competent again, re-transformed with CRIM plasmid, and grown on chloramphenicol plates at 30°C. During this stage, CRIM plasmids were maintained as high-copy plasmids by the *pir116 *gene product expressed from the helper plasmid. From the transformants obtained, a single colony was grown to saturation in 5 mL LB medium supplemented with chloramphenicol at 37°C, and then re-streaked on chloramphenicol plates and grown overnight at 43°C. Following genomic integration of the CRIM plasmid into the target MC1061 *E. coli *strain, growth at this non-permissive temperature cures the cell of the helper plasmid containing the *pir116 *gene. From these colonies, a single colony was re-grown to saturation in 5 mL LB medium supplemented with chloramphenicol at 37°C, and then streaked on ampicillin plates to confirm loss of the helper plasmid. Genomic DNA was then isolated from the resulting cells and analyzed to confirm integration of the CRIM plasmid into the genome. Specifically, the Φ80 locus was PCR amplified with primers CA603F (5'-CTGCTTGTGGTGGTGAAT-3') and CA603R (5'-TAAGGCAAGACGATCAGG-3'), and PCR products were sequenced to confirm the integration of parts.

### Post-integration marker excision from CRIM integrants

Following confirmation of the genomic integration event, the chloramphenicol marker and R6K origin of replication originally carried in the CRIM plasmid were removed from the genome by Flp-mediated recombination. Briefly, competent cells of the integrant were transformed with helper plasmid pCP20 [23] containing Flp recombinase, and grown on ampicillin plates under permissive conditions at 30°C. Following Flp-mediated recombination, a single colony was grown to saturation in 5 mL LB medium lacking antibiotics and then re-streaked onto LB plates and grown overnight at 43°C to cure the helper plasmid. From the resulting clones, a single colony was grown to saturation in LB medium at 37°C, and then re-streaked on ampicillin plates to confirm loss of the helper plasmid. Genomic DNA was then analyzed by PCR and sequencing of the Φ80 *att *locus to confirm loss of the chloramphenicol marker and R6K origin of replication from the host genome.

## List of abbreviations used

CRIM: conditional-replication, integration, and modular plasmids; RBS: ribosome binding site; ORF: open reading frame; BBF: BioBricks Foundation; RFC: request for comments.

## Declaration of competing interests

The authors declare that they have no competing interests.

## Authors' contributions

The BglBrick standard was designed by JCA, JED, JAG, APA, and JDK. The RBS variants, *lacZ *expression devices, and CRIM integration studies were performed by JCA. The protein-protein interaction studies were performed JED and GCW. The manuscript was drafted by JCA, JED and ML. All authors read and approved the final manuscript.
